# Rapid response to emerging biomedical challenges and threats

**DOI:** 10.1107/S2052252521003018

**Published:** 2021-03-26

**Authors:** Marek Grabowski, Joanna M. Macnar, Marcin Cymborowski, David R. Cooper, Ivan G. Shabalin, Miroslaw Gilski, Dariusz Brzezinski, Marcin Kowiel, Zbigniew Dauter, Bernhard Rupp, Alexander Wlodawer, Mariusz Jaskolski, Wladek Minor

**Affiliations:** a Department of Molecular Physiology and Biological Physics, University of Virginia, Charlottesville, Virginia, USA; b College of Inter-Faculty Individual Studies in Mathematics and Natural Sciences, University of Warsaw, Warsaw, Poland; c Faculty of Chemistry, Biological and Chemical Research Center, University of Warsaw, Warsaw, Poland; d Department of Crystallography, Faculty of Chemistry, A. Mickiewicz University, Poznan, Poland; e Center for Biocrystallographic Research, Institute of Bioorganic Chemistry, Polish Academy of Sciences, Poznan, Poland; f Institute of Computing Science, Poznan University of Technology, Poznan, Poland; g Center for Structural Biology, National Cancer Institute, Frederick, Maryland, USA; h k.-k Hofkristallamt, San Diego, California, USA; i Institute of Genetic Epidemiology, Medical University Innsbruck, Innsbruck, Austria

**Keywords:** COVID-19, SARS-COV-2, coronavirus, pandemic, bioreproducibility, information noise

## Abstract

As part of the global mobilization to combat the present pandemic, almost 100 000 COVID-19-related papers have been published and nearly a thousand models of macromolecules encoded by SARS-CoV-2 have been deposited in the Protein Data Bank within less than a year. The avalanche of new structural data has given rise to multiple resources dedicated to assessing the correctness and quality of structural data and models. Here, an approach to evaluate the massive amounts of such data using the resource https://covid19.bioreproducibility.org is described, which offers a template that could be used in large-scale initiatives undertaken in response to future biomedical crises. Broader use of the described methodology could considerably curtail information noise and significantly improve the reproducibility of biomedical research.

## Abstract 2

When biomedical crises strike, structural biologists worldwide respond by determining the structures of relevant proteins and their complexes, resulting in an avalanche of data that can be overwhelming without a resource designed to classify, annotate and validate them. An advanced information system is necessary to extract and infer knowledge from a deluge of uncurated and disjointed data and publications.

## 1. Introduction   

In response to the SARS-CoV-2 pandemic, an unprecedented mobilization of the scientific community took place, focused on understanding various properties of the SARS-CoV-2 virus and developing drugs to treat and prevent COVID-19. In line with the current, structure-based paradigm of drug discovery, structural biology has been among the leading disciplines supporting these efforts. Indeed, since the first structure of a SARS-CoV-2 protein was released at the beginning of February 2020, there has been a flood of three-dimensional models of SARS-CoV-2-related macromolecular targets, mostly determined by X-ray crystallography and cryo-electron microscopy (cryo-EM). The worldwide Protein Data Bank (wwPDB), which is a global repository of experimental macromolecular models, functioning as a consortium of the RCSB (USA), PDBe (Europe) and PDBj (Japan), is essential for making these efforts publicly available (Berman *et al.*, 2000 ▸; Burley *et al.*, 2019 ▸). The urgency to understand the pathological mechanisms of this virus and to find therapies resulted in an extremely rapid pace of research and a large number of structural depositions in a short period of time. This rapidity has inevitably led to mistakes and errors of different severity, sometimes in the sensitive area of protein–ligand interactions, potentially misleading subsequent biomedical research efforts. Such a scenario calls for an additional ‘quality-control’ step that guarantees the validity of the models (Clegg, 2021 ▸; Wlodawer *et al.*, 2018 ▸). Such a step is now an accepted part of the structure-based drug-design protocol. Accordingly, several projects were initiated to assess the SARS-CoV-2 structures (Wlodawer *et al.*, 2020 ▸; Croll *et al.*, 2020 ▸), in addition to previously established structure re-refinement resources such as *PDB-REDO* (Touw *et al.*, 2016 ▸). These efforts resulted in the creation of web servers, for example https://covid-19.bioreproducibility.org, that are meant to organize the workflow and make the results more easily digestible to the biological and medicinal communities (Brzezinski *et al.*, 2021 ▸).

Recently, ∼50 various COVID-19-related resources were described in a publication that may serve as a ‘meta-resource’ (Waman *et al.*, 2020 ▸). However, maintenance of these resources and keeping them up to date is a daunting task, as shown by the number of high-visibility (not necessarily COVID-19-related) resources that have been closed (Berman *et al.*, 2009 ▸) or have became defunct in recent years (Kolesov *et al.*, 2007 ▸). The weekly inflow of new SARS-CoV-2-related structures has not faded away but has remained high (on average about 15 per week), overwhelming even teams of highly experienced structure-validation experts. With the comfort provided by the presence of high-quality structures for most of the viral proteins, it became obvious to us that the long-term role of the above-mentioned resources is not to scrupulously analyze every structure as soon as it appears in the PDB, but to demonstrate a possible path to handle a large amount of structural data during current and future biomedical challenges.

At this point, two observations were made. Firstly, it was gratifying to note that only a small fraction of the crystallo­graphic structures needed moderate corrections, with less than 1% requiring significant reinterpretation. We decided to give the cryo-EM structures only a very superficial inspection, as such detailed correction of structural problems of cryo-EM models would be beyond the scope of this paper. Secondly, as previously reported (Raczynska *et al.*, 2018 ▸), attempts at completely automatic model correction, for example via *PDB-REDO* (Touw *et al.*, 2016 ▸), are limited in scope and cannot address some issues, such as ligand identification and major rebuilding beyond the radius of convergence of simple re-refinement. A human expert is still needed to correct the remaining errors, perhaps assisted by some artificial intelligence (Kowiel *et al.*, 2019 ▸). Another example of a structure-improvement resource is the *Cryo-EM Re-refinement System* (*CERES*) set up by the Phenix group (Liebschner *et al.*, 2021 ▸).

With an accumulation of structures to validate and possibly re-model, re-refine and re-deposit, we decided to create an automatic tool to generate a report for each structure (Brzezinski *et al.*, 2021 ▸) that goes beyond the validation report provided by the PDB. Our aim was to rapidly evaluate which cases need to be manually inspected and possibly corrected. We would like to stress that any improved structure **should be re-deposited** to the PDB by the original authors, sometimes together with those who significantly contributed to structure improvement, to ensure that the PDB contains the most accurate model and that the original authors receive full credit for their work. The versioning scheme recently implemented by the PDB should make this task easier. In cases of disagreement with the original depositors, it may be necessary to make a secondary deposition based on the original authors’ structure factors.^1^ The approach described here can serve as a template for various large-scale initiatives that assess macromolecular structure models as drug-design targets.

## 2. Structure assessment   

### 2.1. Harvesting and classification of structural models   

Since the beginning of the COVID-19 pandemic, almost 100 000 COVID-19-related papers have been published in journals indexed by PubMed, constituting ∼6% of all biomedical papers during that time. Simultaneously (as of 1 February 2021), nearly 1000 macromolecular models related to SARS-CoV-2 have been deposited in the PDB. The depositions comprise numerous models of the same viral proteins, sometimes whole, sometimes their individual domains, and sometimes complexes of various combinations of the viral proteins (or their mutants or domains) and antibodies, nucleic acids or human proteins. There are enough of these combinations to completely overwhelm biomedical researchers who want to utilize these models in their research. Every week, over 200 new macromolecular models (not just COVID-19-related) are deposited in the PDB, and a detailed analysis of this structure flow is a Sisyphean task. To select only structures of interest, we decided to perform a sequence-similarity check for every new PDB deposition with SARS-CoV-2 proteins (an excellent artist’s interpretation of the SARS-CoV-2 virion and its proteins is presented in Parks & Smith, 2020 ▸). This technique was previously used by Protein Structure Initiative centers (Chruszcz *et al.*, 2010 ▸; Grabowski *et al.*, 2016 ▸) to check every week whether other crystallo­graphers or research entities had tackled any of the MCSG, NYSGRC and CSGID target homologs. This procedure reduces the number of potential candidates for scrutiny by 90% and at the same time allows us to properly classify each new SARS-CoV-2-related deposition. The structures are then classified as native, mutants, complexes with smaller ligands or complexes with other macromolecules, which could be antibodies or other biological macromolecules, such as cell-surface receptors or nucleic acids. This information could in principle be extracted from the PDB resource dedicated to COVID-19 (Lubin *et al.*, 2020 ▸; https://www.rcsb.org/news?year=2020&article=5e74d55d2d410731e9944f52), which was indeed our initial method, or from the PDB header and title records, but we decided to verify it by sequence-similarity search, especially when we found discrepancies between our data harvesting and PDB classification. When a given deposition is a ligand–macromolecule complex, we routinely check the the correlation of the ligand model with its electron-density map.

### 2.2. Structure-assessment criteria   

**Figure 1:**
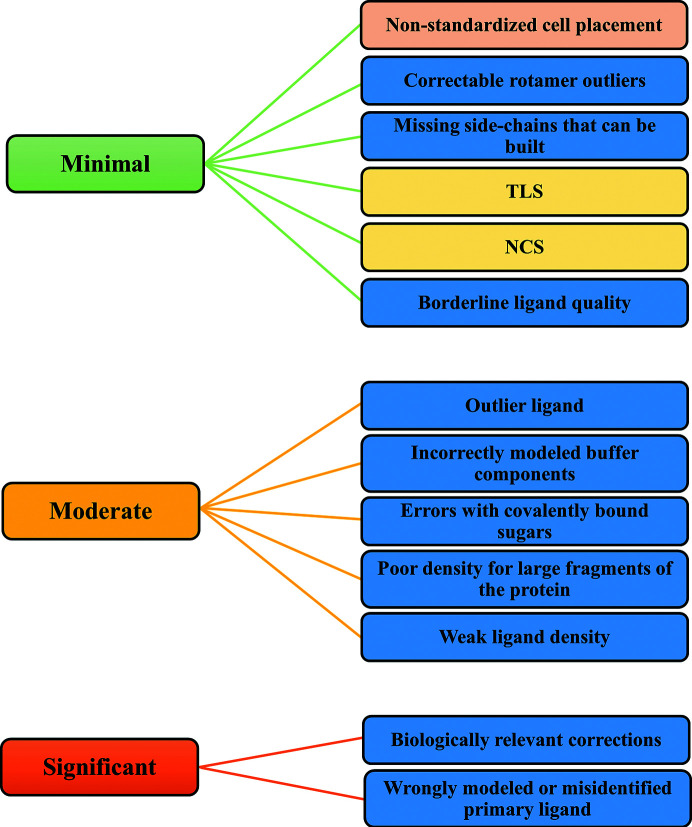
Classification of problems noted in various macromolecular structures. These problems, which are not an exhaustive list, may be difficult or even impossible to correct. Some criteria are case- and resolution-dependent, such as NCS and TLS (indicated in a different color). For example, the use of NCS may be critical for low-resolution structures due to the decreased number of parameters. Nonstandardized cell placement should be avoided because it makes it more difficult to compare two or more similar structures (also indicated by color). The classification may depend on who is looking at the structure, *i.e.* a crystallographer or a biologist.

The criteria that we use to evaluate each structure result from experience acquired over several such projects and are relatively straightforward. They were previously described in the paper introducing the https://covid-19.bioreproducibility.org server (Brzezinski *et al.*, 2021 ▸), as well as in several papers published on this subject (Wlodawer *et al.*, 2013 ▸; Shabalin *et al.*, 2015 ▸; Minor *et al.*, 2016 ▸; Zheng *et al.*, 2014 ▸). For convenience, we broadly classified the issues noticed in structures into three categories: minimal, moderate and significant. These are the terms that we used to triage the COVID-19-related structures according to the perceived need to re-refine a deposited structure. These criteria should be seen as examples, and should not be considered to represent a strict or exhaustive list (Fig. 1 ▸). The importance of each issue is structure- and resolution-dependent, and different researchers may not agree with the ranking of each point in Fig. 1 ▸. However, all of these issues are important and should always be addressed before a structure is deposited. For example, nonstandard placement in the unit cell may be a minimal error if it affects a unique structure, but is more serious if there are already structures in that space group, since it will un­necessarily complicate their comparison.

It is important to realize that the criteria are not written ‘in stone’; crystallographers working on re-refinements may have different opinions on the severity level of the same issue. New experiences in structure re-determination may improve the process and may affect the point of reference. The large number of essentially very similar structures elucidated in a very short period creates an opportunity to compare various quality metrics and the influence of crystallization conditions, and also to analyze how different space-group polymorphs might affect structure interpretation.

**Figure 2:**
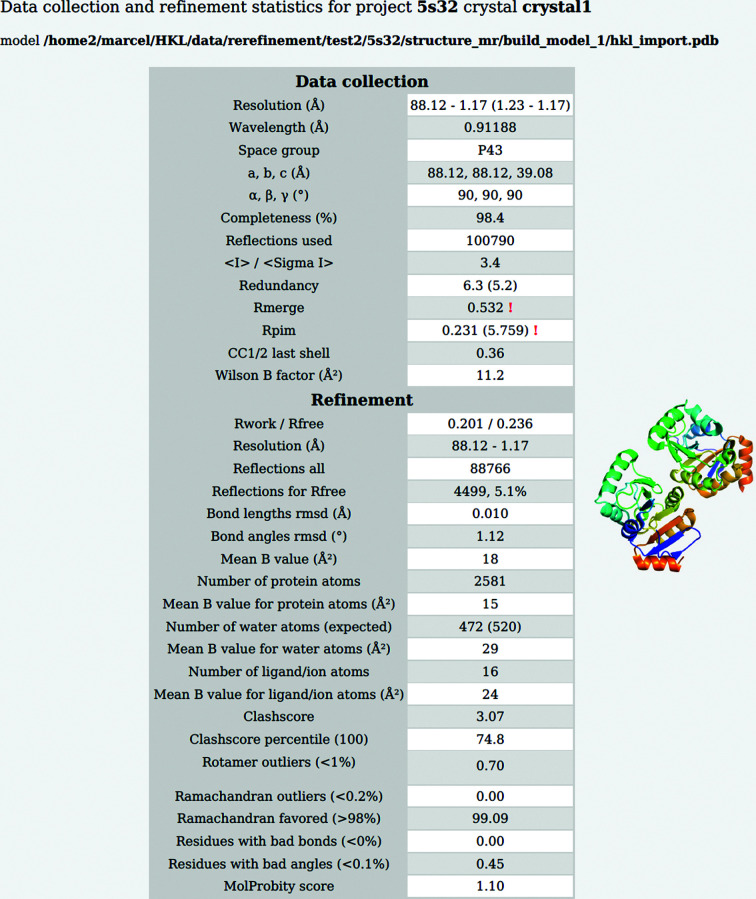
Part of a preliminary report showing the most important parameters related to structure quality, as exemplified by deposition 5s32 imported from the PDB. Unusual, missing or questionable values are flagged with red exclamation marks. The full report is presented in Supplementary Fig. S1.

Each model is downloaded from the PDB and analyzed by *HKL*-3000 (Minor *et al.*, 2006 ▸), and the results are presented in an updated format (Fig. 2 ▸) of a standardized report of data and model quality (Brzezinski *et al.*, 2021 ▸). Unusual, missing or questionable values are flagged with red exclamation marks. When a structure is re-refined, the new report contains the results of re-refinement; additionally, it may include re-processing results if the original diffraction data are available in a data repository. Some repositories check the consistency between the diffraction data and structural models submitted to the PDB. For example, in the IRRMC resource at https://proteindiffraction.org, all diffraction data are automatically re-processed to verify that the correct data are associated with each structure (Grabowski *et al.*, 2016 ▸, 2019 ▸). It is important to note that roughly 5% of the original data deposited in the IRRMC were initially inconsistent with the corresponding PDB deposition, which shows that data management in crystallo­graphic laboratories is still in need of improvement (Zimmerman *et al.*, 2014 ▸; Cooper *et al.*, 2021 ▸). Metadata that are only contained in the PDB itself can be unreliable because they are supplied by the researcher who made the deposition. Inexperience or haste may lead to information being submitted to the wrong field, to inappropriate values being entered or to data items being skipped. First-time depositors make as many as 20% of all PDB depositions (assuming that the first author of a structure is responsible for the deposition); therefore, mistakes are not uncommon.

**Figure 3:**
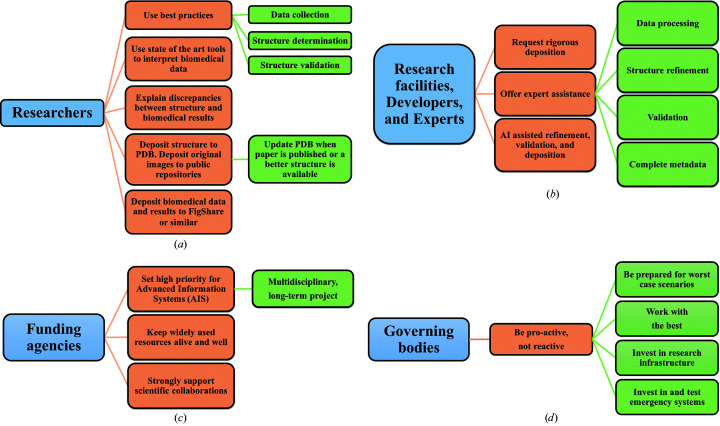
Rapid response will happen only when researchers, facilities, funding agencies and governing bodies work together. Our recommendation is just a voice in the discussion and, as such, is highly subjective.

To address the issue of metadata integrity and the subsequent reproducibility of biomedical research, we make a number of suggestions, presented in Fig. 3 ▸. In our opinion, these suggestions should be implemented on many levels (researchers, data and research facilities, funding agencies and governing bodies) in a coordinated fashion. In addition, we believe that it is crucial for journals to require the deposition of all relevant structures before submission of a paper and the provision of the reviewers of the paper with the structure and the corresponding electron-density maps. As an absolute minimum, the reviewers should be provided with all of the information that is submitted to the PDB.

### 2.3. Importance of the availability of original diffraction data   

The reproducibility and independent validation of structural models strongly rely on the availability of the primary experimental data. For X-ray crystallography, the primary data are a set of diffraction images. For cryo-EM, the primary data are a set of electron-microscopy images. The process of 3D structure determination involves multiple transformations of these sets of images, generally resulting in a reduction of the size of the data files and in the potential loss of some information. In a typical X-ray structure-determination pipeline, the structure-factor amplitudes are extracted from individual reflection intensities that are scaled and merged by data-processing software (Otwinowski & Minor, 1997 ▸; Minor *et al.*, 2006 ▸; Leslie, 2006 ▸; Kabsch, 2010 ▸; Winter *et al.*, 2018 ▸). The resulting reduced form of the data is saved in ‘structure-factor files’ containing structure-factor amplitudes, which are deposited in the PDB along with the atomic coordinate files.

Historically, the original diffraction data were often lost or discarded due to their size exceeding the limited storage space. The structure model was thought to be the ultimate result of a crystallographic experiment, and access to the coordinates (and later to the structure factors as well) was considered to be sufficient. However, a lack of access to full diffraction data makes it impossible to validate the data-processing step. In our experience, there have been multiple cases in which re-processing the original data has dramatically improved the resolution and/or quality of an already deposited structure (Shabalin *et al.*, 2015 ▸). The importance of archiving primary diffraction data has been underscored by IUCr Journals in a joint editorial (Helliwell *et al.*, 2019 ▸). To archive their diffraction data, crystallographers now have specialized repositories at their disposal, with SBGRID (Meyer *et al.*, 2016 ▸) and IRRMC (Grabowski *et al.*, 2019 ▸) being the most important. In addition, several general-purpose data repositories such as Zenodo (https://zenodo.org/) and Figshare (Singh, 2011 ▸) can be used; however, the general-purpose repositories are usually unstructured and accept data ‘as is’. For cryo-EM images, the EMPIAR (Iudin *et al.*, 2016 ▸) resource is available. However, so far only a small percentage of primary data for COVID-19-related structures are publicly available. As of 1 February 2021 the IRRMC contained 41 X-ray data sets for SARS-CoV-2 proteins and three data sets for the related coronavirus H-CoV-229E. Several more were available at SBGRID. EMPIAR contained 14 sets of SARS-CoV-2-related cryo-EM images. Zenodo contained 79 data sets for the 3CLpro main protease, 78 of which represent a single PanDDA deposition group.

Considering the increased use of preprint servers, such as medRxiv and bioRxiv, the ASAPbio organization has recommended that scientists do not wait until the paper is published in a peer-reviewed journal to release their PDB depositions, but do so at the time when it becomes publicly available as a preprint (https://asapbio.org/asappdb). Scientists are also encouraged to deposit primary experimental data, such as diffraction data, to one of the dedicated resources.

In most cases, the structure factors deposited in the PDB are sufficient for an initial structure validation. However, when access to the original diffraction images is necessary and they are not publicly available, the only way to obtain the data is to ask the authors. If a publication is associated with the deposition, then it is possible to use the email address of the corresponding author and/or ask the journal to request primary data from the authors. However, contacting the depositors becomes complicated when a deposition does not have a primary citation and thus no depositor contact information is available. This obstacle makes PDB entry validation, or any task requiring communication with the original authors, unduly challenging, and a substantial number of our requests for diffraction data have gone unanswered. The reluctance to provide primary data is not unique to structural biology. It was recently reported that a requirement of access to primary data resulted in a dramatic reduction in accepted manuscripts (Miyakawa, 2020 ▸). Since 2007, the deposition of structure factors has been mandatory; nevertheless, requests for biomedical data or diffraction images are sometimes ignored. It seems that a similar requirement, *i.e.* deposition in a public database, if made by all structural biology journals, would greatly reduce information noise and significantly improve bioreproducibility. As of 1 February 2021, 360 out of 930 SARS-CoV-2 depositions had a primary citation listed in the PDB. The rest (including 286 non-PanDDA^2^ structures) remain ‘to be published’.^3^ A significant fraction of the primary citations published so far were in high-impact journals.

### 2.4. Inconsistent annotations   

**Figure 4:**
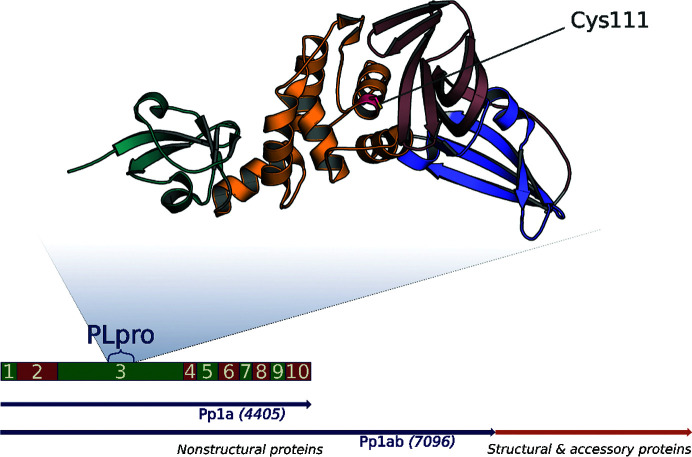
Location of the PLpro gene in the SARS-CoV-2 genome. The red and green rectangles indicate the nonstructural proteins (NSPs). The N-­terminal ubiquitin-like, thumb, zinc-fingers and palm subdomains are colored teal, orange, raspberry and blue, respectively, in the cartoon model of PDB entry 6wx4. The catalytic Cys111 residue is marked in red.

During the protein-classification step, we encountered a number of problems with inconsistent nomenclature and annotations. These problems can be illustrated by the example of papain-like cysteine protease (PLpro) structures (Fig. 4 ▸). PLpro is an essential enzyme necessary for the proteolysis of the replicase complex and is a promising target for drugs inhibiting virus replication (Báez-Santos *et al.*, 2015 ▸). In all known coronaviruses, the PLpro domain, a member of the PFAM08715 family, resides inside the NSP3 region (Lei *et al.*, 2018 ▸), albeit at different locations. In SARS-CoV-2 the PLpro domain spans residues 1564–1882 of the multiprotein Orf1ab (746–1064 of NSP3), in MERS-CoV residues 1484–1800 of Orf1ab (631–947 of NSP3) and in SARS-CoV residues 1541–1859 of Orf1ab. Because of difficulties in crystallizing wild-type PLpro, a Cys→Ser mutation that inactivates the catalytic triad is often used to facilitate crystal growth. This technique was first applied for the SARS-CoV virus, yielding the structure of the C112S PLpro mutant (PDB entry 4m0w; Chou *et al.*, 2014 ▸). In the SARS-CoV-2 virus the corresponding mutation is made at position 111 of PLpro, and the PDB now contains multiple structures of this C111S mutant. Confusingly, some structures describe this mutation incorrectly as occurring at position 112 of PLpro (for example, PDB entries 7d6h and 7d7t; J. Liu, Y. Wang & L. Pan, unpublished work). The ‘Protein Feature Viewer’ on the PDB webpage shows the mutation at variable positions because the sequence is numbered from the beginning of the modeled structure (Table 1 ▸) instead of using a fixed reference sequence. This inconsistency in denoting the mutation site may seem to be a minor issue, but it creates the potential for confusion in the interpretation of structures by noncrystallographers and/or by automated tools, especially when the structure is released without an associated paper. Fortunately, careful users of the PDB viewer can resolve the confusion by looking to the absolute position of the mutation within ORF1ab, which disambiguates the position of the mutation in the PDB file.

The differences in residue numbering between various PLpro structures are a source of frustration that is not limited to this project and can significantly complicate structure analysis and data mining. Residue numbers should conform to some standard, and using a mixture of numbering methods within a protein family unnecessarily complicates structural comparisons. This problem was recently addressed by the *PDBrenum* web server, which provides structures that have been renumbered according to their UniProt sequences (Faezov *et al.*, 2021 ▸). However, without general acceptance of this convention by the PDB, the files resulting from this server could ultimately contribute to the very confusion that it aims to alleviate.

### 2.5. Inconsistencies within PDB depositions   

During our attempts to generate automatic reports and re-process the diffraction data, we encountered a major issue with PDB depositions that is of a general nature and calls for a revision, or at least an inspection, of the deposition/reporting system used by the wwPDB. According to the declaration of the wwPDB, any data deposited using the universal OneDep tool should be consistent and identical regardless of which PDB site was used for deposition. However, not all of the information presented on the websites of the three organizations that collect and disseminate information as part of the wwPDB (*i.e.* RCSB PDB, PDBj and PDBe) is identical. For example, the 〈*I*/σ(*I*)〉 values reported for deposition 6zh9 appear to be different on the PDBe site, as illustrated in Supplementary Fig. S2. The PDBe information is not based on the mmcif entry but seems to be based on the results of a *phenix.xtriage* analysis of the deposited structure factors.

## 3. Case studies   

As of 1 February 2021, the covid19.bioreproducibility.org resource identified minor or moderate quality issues in about 100 structures and significant issues in nine structures. One of these, PDB entry 7d1m, has been re-deposited (Brzezinski *et al.*, 2021 ▸). Some of the structures in which we found quality issues (PDB entries 6w41 and 6w9c) have also been identified by other structure-assessment resources (Croll *et al.*, 2020 ▸, 2021 ▸). To illustrate our approach, we present two case studies analyzing the set of structures of two subdomains of NSP3: PLpro and the macrodomain.

### 3.1. Case study 1: comparative analysis of SARS-CoV-2 PLpro structures   

As of 1 February 2021, the PDB has released 25 deposited structures of PLpro from SARS-CoV-2. These depositions represent either the structure of the protease in isolation, in complex with small-molecule ligands (candidate inhibitors) or in complex with other proteins, for example ubiquitin-like protein.

All of these crystal structures were determined using X-ray diffraction on different beamlines, by ten research groups, with molecular replacement (MR) utilized for structure solution. The CSGID determined the first of these structures, PDB entry 6w9c (deposited on 22 March and released on 1 April 2020). It used MR based on the structure of PLpro from the previously studied SARS-CoV virus (PDB entry 5y3q). The second structure (PDB entry 6wrh) was released about a month later for the C111S mutant, and together these two structures have been used as the starting MR models for 17 of the subsequent 23 PLpro structures (some of the subsequent structures of PLpro complexes used multiple models). 13 structures of PLpro were determined by the CSGID and have the same first author, who also identified several small-molecule inhibitors (Osipiuk *et al.*, 2021 ▸). The PLpro structures in the PDB contain seven unique inhibitors bound to the enzyme (Supplementary Fig. S3).

**Figure 5:**
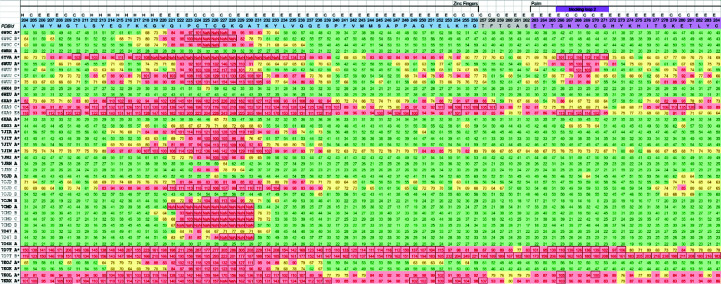
Comparison of residue *B* factors (Å^2^) in all known X-ray structures of PLpro from SARS-CoV-2, identified by PDB code on the left. A green color indicates values below the average for all atoms of all PLpro structures and yellow indicates values that are above. Residues with a *B* factor greater than 80 Å^2^ are marked in red, and a red frame indicates values that are more than one standard deviation higher than the average. An asterisk by the PDB code indicates that TLS was used during refinement. The secondary structure is assigned per residue by a one-letter code at the top of the figure: C, E and H, representing coil, strand and helix, respectively. ‘Blocking loop 2’ is marked in purple. The full spreadsheet is available as supplementary data.

Analysis of the deposited structures of PLpro from SARS-CoV-2 shows a wide distribution of the quality of the models. One way of assessing the quality is through analysis of the ADP (or *B*-factor) distribution (Rupp, 2009 ▸; Masmaliyeva & Murshudov, 2019 ▸) within the structures and among them (Fig. 5 ▸). Generally speaking, higher ADP values mean lower precision of the atomic positions. However, when comparing ADPs between structures, it is important to keep in mind that somewhat higher ADPs do not necessarily mean a less accurate structure, in part because ADP distributions are dependent on the ADP restraint implementation of the refinement. In particular, it is important that when translation–libration–screw (TLS) refinement was used in *REFMAC* that the full atomic anisotropic displacement tensor values are deposited, because in the atom records only the residual *B* factor is listed (https://www.wwpdb.org/deposition/refmac-user-notice). The anisotropy records (or their restoration from the TLS records) are necessary to reconstruct the full *B* factor. The analysis of the ADPs in Fig. 5 ▸ shows a very similar pattern among the SARS-CoV-2 PLpro models, in which the loops between the major secondary-structure elements have higher ADPs than the core of the protein. Some other high-motion regions are common to multiple structures, in particular ‘blocking loop 2’ (Gly266–Gly271) within the palm subdomain (Henderson *et al.*, 2020 ▸). Other flexible regions occur within the zinc-fingers subdomain (Fig. 4 ▸).

**Figure 6:**
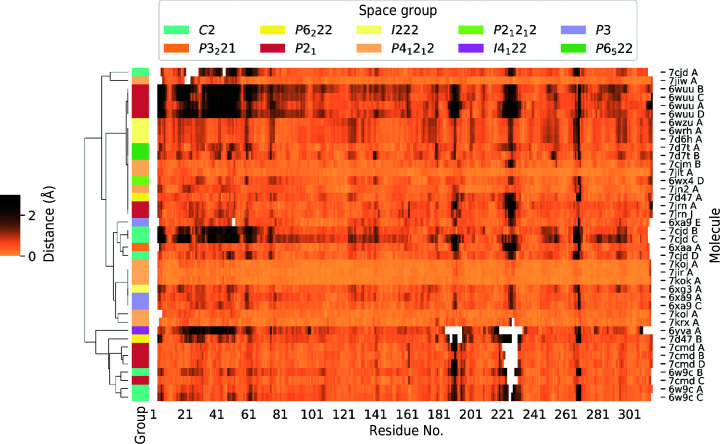
A map showing the distances between equivalent C^α^ atoms (numbered at the bottom) in a given PLpro model (identified by PDB code and chain ID on the right) and the reference structure PDB entry 7yvi selected as the medoid model (see the text for an explanation). A white color indicates residues that are missing in a given model. The dendrogram on the left shows the results of clustering using Ward’s method (Ward, 1963 ▸).

The regions of high flexibility can also easily be glimpsed from an inter-structure distance map (Fig. 6 ▸) as regions with the highest deviations from the medoid structure (PDB entry 7yvi). The medoid structure was selected as the model with the smallest r.m.s.d. from all complete (no missing residues) PLpro models. It is easy to single out problematic residues from the contact map (Supplementary Fig. S5). This is an example of analysis that can be performed for an ensemble of similar structures. Both maps were calculated using *BioShell* (Macnar *et al.*, 2020 ▸).

**Figure 7:**
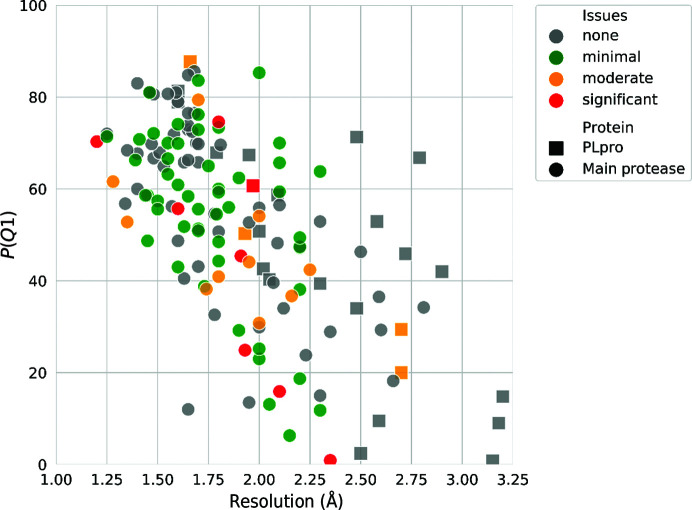
Comparison of protein structure resolution and an overall structure-quality indicator, *P*(*Q*1), which combines *R*_free_, RSRZ (normalized real-space *R*-factor) outliers, Ramachandran outliers, rotamer outliers and clashscore into a percentile scale (Brzezinski *et al.*, 2020 ▸). The comparison involved models of PLpro (squares) and 3CLpro (circles). Higher values of *P*(*Q*1) represent better models. The colours indicate the severity of the problems detected by the https://codvid19.bioreproducibility.org server. An important lesson for all docking and/or computational studies is that structure quality does not depend on resolution only (see Supplementary Fig. S4).

Of all the PLpro structures that we analyzed, only one had significant quality issues (see below), while four had moderate issues. In comparison, 11 structures of the main protease had moderate corrections, while seven had significant errors (Fig. 7 ▸). The first deposited SARS-CoV-2 PLpro structure, PDB entry 6w9c, is classified as having moderate quality issues. It was determined at 2.7 Å resolution, with most of the residues having very high ADPs. Many residues were found in extremely poor electron density. It is important to note that these issues with structure quality are not a result of poor refinement but rather of low-quality data (completeness of 57.3%) caused by radiation damage. Re-refinement of PDB entry 6w9c with added noncrystallographic symmetry (NCS) restraints for the three independent copies of the molecule in the asymmetric unit fixed a number of rotamer outliers, but it could not substantially improve the model due to poor electron density.

As this structure was the first deposited model of the papain-like protease of SARS-CoV-2, there was understandably a rush to deposit this structure to make it available to the scientific community. In terms of lessons from this pandemic, we think that rapid but imperfect deposition is a winning strategy. Even though the first structure was poor, it still provided a good idea about the fold of the protein and the details of the active site, confirming its similarity to the SARS-CoV homolog, and contributed to the determination of subsequent PLpro structures. Later, structures of the same protein with much better quality were released by the same authors and by others, and these should be used at present by anyone working on this subject.

Three other PLpro structures were identified to have moderate quality issues: missing a few amino-acid residues, side chains or water molecules, or with incorrect rotamers, water molecules marked as UNK *etc*. Re-refinement was able to fix most of these issues. One structure (PDB entry 7d47), which originated from a twinned crystal, was classified as having significant quality issues: the coordinates were not in the standardized location in the unit cell and several residues were missing in chain *B*. Even though the electron density in the area of the missing residues was not very strong, it was possible to trace the amino-acid residues and add them during re-refinement. Additionally, NCS restraints were used during re-refinement and several water molecules were added to the model.

While the changes introduced during re-refinement may appear to be relatively minor and inconsequential, one has to take into account the possibility that inferior structure quality may impact subsequent studies. Targeting PLpro with small-molecule inhibitors is a promising anti-COVID strategy that has been already explored by several docking studies (Rahman *et al.*, 2021 ▸; Hall-Swan *et al.*, 2021 ▸; Sedova *et al.*, 2020 ▸). However, it appears that these docking studies did not use the curation/re-refinement results provided by any of the quality-assessment resources for SARS-CoV-2 structures. Some of the docking studies relied on structures for which moderate quality issues were identified by our resource, such as PDB entry 6w9c. This illustrates a significant limitation of assessment projects, namely that structural improvements that are not reported to the PDB can have only a limited, if any, impact on subsequent research. There is no doubt that many of the models in the PDB-REDO databank are better than the original PDB depositions; however, the improved structures are used much less frequently than those from the PDB. Analysis of literature references shows that the number of citations of *PDB-REDO* is more than two orders of magnitude lower than that of the PDB. For this reason, when significant changes are necessary, the authors of this paper always follow the path to joint depositions, as described in Section 1. In the majority of cases, we strongly encourage the authors of the original depositions to **make use of our corrections and update the models in the PDB using the recently implemented versioning mechanism**, which allows depositors to update their entries while retaining the same PDB accession code. Moreover, as nearly all publications are now available online, it would be beneficial if the update to the PDB deposition or a link to the new PDB code (if the structure was re-deposited due to updated structure factors) could also be added as a note to the original publication. We encourage either taking the updated models available on our website as a starting point or simply using the list of corrections in the ‘Re-refinement summary’ for each structure. As these models are not always fully finalized, all corrections should be inspected by the authors, new PDB validation reports should be run and any remaining issues may need to be addressed. The resource provides a contact email for questions regarding particular corrections.

There is an important issue created by the presence of older suboptimal structures in the PDB. Sometimes the same group reports a new and better structure, but for various reasons the older one is still left in the PDB. When suboptimal structures are used in docking studies, the docking is also suboptimal or may even be plainly wrong. However, this observation leads to a question: should the earlier structure be retracted from the PDB, in order to reduce ‘pollution’ of the database, or should it stay there as a historical record of a landmark and timely achievement of the authors? If the latter is chosen, there should be a flag (keyword) warning about the use of such historical depositions for subsequent studies, and redirecting to the superseding deposition. Perhaps the PDB interface can adopt Amazon’s approach and display the message ‘A newer version of this structure is available’.

### 3.2. Case study 2: atomic resolution structures of the SARS-CoV-2 NSP3 macrodomain   

A large number of structural models of the SARS-CoV-2 NSP3 macrodomain determined at atomic resolution (1.2 Å or higher) have been deposited during the last year. These models were deposited by two different groups (126 by J. S. Fraser and coworkers, and 100 by F. von Delft and coworkers). A vast majority of these structures are annotated as group depositions, although not all are clearly identified as members of PanDDA sets. Some structures, however, were deposited individually and not in groups (examples include PDB entries 7kqw, 7kqo, 7kr0 and 7kqp). Considering that such atomic resolution structures are commonly used for follow-up in-depth studies and may be used for the creation of accurate restraint parameters for the refinement of protein structures at lower resolution (Jaskolski *et al.*, 2007 ▸; Jaskolski, 2017 ▸), it is crucial that they are refined with particular care and properly annotated during deposition. This, however, does not appear to be the case here.

**Figure 8:**
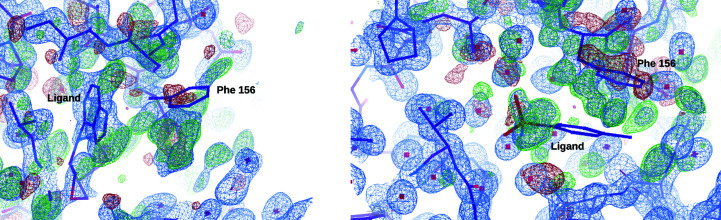
Two examples of PanDDA structures. Left, deposition 5rtl; right, deposition 5rsi. The models are shown in stick representation with C atoms in magenta, O atoms in red and N atoms in blue. The maps and models are as downloaded from the EDS server, as these are those that a regular user would use. The electron-density maps are contoured at 1.0 r.m.s.d. for 2*mF*_o_ − *DF*_c_ (blue) and ±3.0 r.m.s.d. for *mF*_o_ − *DF*_c_ (green/red). The maps clearly show that in both structures the Phe156 residue is out of density. While there is some density for the ligand in 5rtl (left), the density does not support the ligand at all in 5rsi (right). The maps and models can be inspected interactively at https://molstack.bioreproducibility.org/project/view/UpsJDYBUP96ULQ63VEUW/.

A large part of the problem is due to the lack of a clear description of what exactly is deposited for each structure in a PanDDA group deposition. Whereas a detailed analysis of the *PanDDA* algorithm is beyond the scope of this paper, we point out that some statistics of such depositions are considerably worse than what would be expected for structures refined at such a high resolution. In particular, the *R* factors are in most cases high, with *R*_free_ mostly above 20%. Unreasonable values of *R*_merge_ (for example, 53% for PDB entry 5s32) are not necessarily the result of a typographical error during deposition, as *R*_p.i.m._ is also very high. What concerned us most, however, were the discrepancies between the atomic coordinates and the electron-density maps calculated using the map coefficients in mtz format downloaded from the RCSB server of the PDB. Two such examples are shown in Fig. 8 ▸ for data sets 5rtl and 5rsi. Whereas the lack of convincing electron density for the modeled ligand may be a feature of the PanDDA approach, the presence of strong electron density for the adjacent protein side chains that does not correspond to the model coordinates is quite troubling.

Another potential problem that we could identify in the individually deposited structures is a very liberal use of multiple conformations in the models, with alternate atomic positions sometimes only as little as 0.1 Å apart. An example is provided by the ultrahigh-resolution structure PDB entry 7kr0, which was modeled with a total of 1995 non-H atomic sites in the protein part. A much more conservative model containing just 1405 sites increased *R*_free_ only minimally, with no significant repercussions in the electron-density map.

## 4. From data to knowledge   

There are two perspectives concerning the value of scientific contributions to combating the COVID-19 pandemic. On the one hand, scientists have produced an avalanche of publications and macromolecular structure models related to COVID-19. On the other hand, all of these efforts have not yet resulted in a definitive cure for the disease. It is possible that some of the published papers contain a blueprint for a cure, but it is very difficult to evaluate the content and importance of each paper among the ∼100 000 published.

In recent years, new ‘science assistant’ tools that use artificial intelligence (AI) to assist humans in the task of identifying and evaluating scientific literature have appeared, with examples such as Scite.ai and Iris.ai. Searching Scite.ai for the ‘PLpro’ keyword identifies 194 publications, displays context and allows citation tracing. Given the URL of a publication, the Iris.ai platform constructs an ‘exploration map’ displaying the concepts appearing in the paper. However, the ‘science assistant’ tools are not yet mature enough to substantially help in finding the most relevant information that may be hidden behind the thousands of pages in dozens of journals. Moreover, there is no connection between these platforms and various important resources, including the structural biology data in the PDB.

We believe that a most promising solution to information overload and the lack of effective information retrieval is the creation of an advanced information system (AIS) (Zheng *et al.*, 2017 ▸) that is capable of harvesting the basic results from all relevant resources and publications. The PDB should be the foundation of a structural biology AIS. This would require a significant improvement of structural depositions, not limited to model coordinates but also emphasizing accurate metadata for each deposition. The first step would be a better definition of the deposition standard, for example the elaboration of guidelines on how to describe areas of the maps that are so weak that one cannot reliably model side chains or even the main chain. Currently, each research group uses their own standard (such as zero occupancy or omitting atoms), and sometimes the same group uses different standards depending on which researcher is responsible for a particular project.

The reliability of scientific data is of paramount importance in many fields. The current biomedical crisis should motivate scientists and science managing bodies to pay more attention to data. The experience with data from over 1000 registered clinical trials for COVID-19 gave rise to the following strong statement (Ewers *et al.*, 2021 ▸):

In these difficult and rapidly changing circumstances, good scientific practice, reproducibility, and transparency are essential principles that must guide clinical trials to adequately inform medical decision-making and keep public trust.

We believe that ‘good scientific practice, reproducibility, and transparency’ should also be the guiding principles of every scientific field, and not only during a health crisis.

To implement these guiding principles, experimental pipelines need to encompass versatile laboratory information management systems (LIMS) to collect complete metadata that are reliable enough to produce the key features of the methods section of the associated publication or, in the case of structural biology, the header of the PDB deposition. Machine-assisted transcription of the metadata is far from viable at this point, but when it becomes available it will have to rely on complete and accurate metadata. Under such a system, if the methods section needs editing, it will mean that the metadata provided for the deposition are not good enough or the routine that produces the methods section is not perfect. Currently, obtaining accurate description of sample preparation for X-ray and cryo-EM experiments from a PDB deposition is particularly challenging. For example, the crystallization conditions included in the PDB file quite often differ from the experimental methods description in the associated publication or lack essential information. Repeating a crystallization, either in a different laboratory or even within the same laboratory, is sometimes a tall order. The creation of an AIS requires a change of attitude: PDB deposition cannot be treated as an obligatory nuisance that is required for publication, but rather as an equally important contribution to the reproducibility and reliability of the permanent scientific record. Achieving such an attitude change may necessitate wider changes in the way that scientific institutions and funding agencies operate. In particular, decisions about hiring, promotion and funding should consider scientists’ contributions to data resources. In other words, the paradigm ‘publish or perish’ needs to be updated to ‘publish good data and papers, or perish’. Otherwise, the pandemic environment may create a ‘publish and still perish’ situation.

The creation of an AIS requires the large-scale collaboration of people with diverse expertise and backgrounds: chemistry, physics, computer science, artificial intelligence, biology, medicine and public policy. The establishment and wellbeing of an AIS should be the joint responsibility of scientists, funding agencies and policy makers.

## 5. Conclusions   

As has happened many times in human history, it turned out once again that a virus, an infectious agent too small to be observed with any light microscope, could shake our advanced civilization by wrecking our economy and disrupting our daily life. SARS-CoV-2 has so far caused the death of more than two million people worldwide and brought some healthcare systems to the brink of collapse, either due to the overflow of COVID-19 patients or, ironically, due to the financial devastation caused by the lack of patients for hospital visits and elective medical procedures due to COVID-19 restrictions. After one year, there are several vaccines in production worldwide, but the logistics of distribution and administration of vaccination is well behind peoples’ expectations and governments’ promises, although some jurisdictions have been able to drastically increase their vaccination efficiency in a short time (Supplementary Fig. S6). At the same time, using a variety of approaches, some countries have been able to significantly reduce the threat of the virus well before the approval of any vaccines. In our opinion, the success of some of these countries, such as New Zealand, Iceland, Finland and Taiwan, can be attributed to science- and technology-savvy leaders who swiftly applied unorthodox thinking to fight the pandemic. Scientific response to the COVID-19 pandemic has resulted in massive amounts of papers, clinical and research data, and structural models, which no single human being can analyze. In 2020, scientists started to create a large number of web resources to help researchers navigate through the COVID-19-related data. However, so many resources have emerged that a meta-resource to these resources has already recently been created (Waman *et al.*, 2020 ▸).

The conversion of mountains of papers and a plethora of structures into useful information is a formidable challenge even in the 21st century. For example, bacterial genomes can now be sequenced quickly and relatively cheaply, but gaining insight into the influence of the individual proteins in the sequenced organism on human health is much more challenging, time-consuming and expensive (McPherson, 2009 ▸). The effective transformation of information and data into knowledge is very challenging and will require a new approach to resources and databases, for example by creating advanced information systems (AISs; Zheng *et al.*, 2017 ▸; Zimmerman *et al.*, 2014 ▸; Cooper *et al.*, 2021 ▸). An AIS will invariably have a database at its core, but will also have a sophisticated system of connections to acquire data from disparate sources (resources and databases) to provide as complete a picture as possible. Creating an AIS will undoubtedly require the collaboration of many scientists who are experts in their respective fields, but it seems to be the only way to prepare biomedical science for the next pandemic.

Within structural biology, many obstacles must be overcome before such an AIS resource can be created, but our experiences can provide guidance to those who would undertake such an endeavor. Structures produced by various laboratories must have a standard evaluation procedure to ensure that they are accurate and conform to accepted standards. This can partially be addressed by the implementation of versioning by the PDB, which will allow structures to be revised when improvements are deemed necessary and can facilitate a more straightforward comparison of related structures. It is essential that discrepancies in the underlying data be fixed when discovered. This is perhaps more important than making a revision to a publication, because coordinates are often used for various purposes (MR models, docking studies, data mining *etc.*) by people who rarely study the original publications and are less likely to routinely search for possible corrections to a publication.

In 2002–2003, a life-threatening SARS-CoV virus with an ∼10% fatality rate infected thousands of people. In 2012, Middle East respiratory syndrome coronavirus (MERS-CoV), with a 43% fatality rate, was identified. Over 13 000 scientific papers on coronaviruses and the related SARS and MERS diseases have been published in the period 2002–2019. Some of these findings strongly suggested the possibility of a future re-emergence of even more deadly outbreaks of SARS-like viruses; nevertheless, an appeal for urgent studies of these viruses (Chou *et al.*, 2014 ▸) went almost unnoticed. Advanced studies of vaccines against the SARS-CoV virus were terminated due to lack of funding (Chen *et al.*, 2014 ▸). In the history of humanity, the COVID-19 pandemic is relatively mild by comparison with the bubonic plague (Black Death) that killed a hundred times more people. **We might not be so lucky next time.**

## 6. Abbreviations   

ADP, atomic displacement parameter; CSGID, Center for Structural Genomics of Infectious Diseases; cryo-EM, cryo-electron microscopy; IRRMC, Integrated Resource for Reproducibility in Macromolecular Crystallography; MCSG, Midwest Center for Structural Genomics; MERS, Middle East respiratory syndrome; MR, molecular replacement; NCS non-crystallographic symmetry; NYSGRC, New York Structural Genomics Research Consortium; PanDDA, pan-dataset density analysis; PDB, Protein Data Bank; r.m.s.d., root-mean-square deviation; RCSB, Research Collaboratory for Structural Bioinformatics; SARS, severe acute respiratory syndrome; TLS, translation–libration–screw.

## Supplementary Material

Supplementary Figures. DOI: 10.1107/S2052252521003018/be5288sup1.pdf

Full spreadsheet corresponding to Figure 5. DOI: 10.1107/S2052252521003018/be5288sup2.xlsx

## Figures and Tables

**Table 1:** Variability in the annotation of the mutation position in structures of the C111S PLpro mutant

PDB code	Title	Position in Feature View	Position in polyprotein	Polyprotein UniProt entry	UniProt ID
6wrh	C111S	114	1674	R1AB_SARS2	P0DTD1
6yva	C111S	111	1674	R1A_SARS2	P0DTC1
6xg3	C111S	114	1674	R1AB_SARS2	P0DTD1
7jir	C111S	114	1674	R1A_SARS2	P0DTC1
7jit	C111S	114	1674	R1A_SARS2	P0DTC1
7jiv	C111S	114	1674	R1A_SARS2	P0DTC1
7cjd	C111S	112	1674	R1AB_SARS2	P0DTD1
7cjm	C111S	112	1674	R1AB_SARS2	P0DTD1
7d47	C111S	111	1674	R1A_SARS2	P0DTC1
7d6h	C112S	116	1674	R1A_SARS2	P0DTC1
7d7t	C112S	116	1674	R1A_SARS2	P0DTC1
7koj	C111S	114	1674	R1A_SARS2	P0DTC1
7kok	C111S	114	1674	R1A_SARS2	P0DTC1
7krx	C111S	114	1674	R1A_SARS2	P0DTC1

## Footnotes

1 A new model that is based on existing original data (structure factors) will only be released by the PDB when submitted together with the original depositors, or when the new model is supported by a peer-reviewed publication. We always prefer to make an updated deposition together with the original depositors.

2 PanDDA (pan-data-set density analysis) deposition groups are related to multi-data-set crystallographic analyses for the identification of ligand binding and structural events (Pearce *et al.*, 2017 ▸).

3 The ‘to be published’ designation is somewhat misleading, because many decades-old structures are still listed as such.

## References

[bb1] Báez-Santos, Y. M., St John, S. E. & Mesecar, A. D. (2015). *Antiviral Res.* **115**, 21–38. 10.1016/j.antiviral.2014.12.015 PMC5896749 25554382

[bb2] Berman, H. M., Westbrook, J., Feng, Z., Gilliland, G., Bhat, T. N., Weissig, H., Shindyalov, I. N. & Bourne, P. E. (2000). *Nucleic Acids Res.* **28**, 235–242. 10.1093/nar/28.1.235 PMC102472 10592235

[bb3] Berman, H. M., Westbrook, J. D., Gabanyi, M. J., Tao, W., Shah, R., Kouranov, A., Schwede, T., Arnold, K., Kiefer, F., Bordoli, L., Kopp, J., Podvinec, M., Adams, P. D., Carter, L. G., Minor, W., Nair, R. & Baer, J. L. (2009). *Nucleic Acids Res.* **37**, D365–D368. 10.1093/nar/gkn790 PMC2686438 19010965

[bb4] Brzezinski, D., Dauter, Z., Minor, W. & Jaskolski, M. (2020). *FEBS J.* **287**, 2685–2698. 10.1111/febs.15314 PMC7340579 32311227

[bb5] Brzezinski, D., Kowiel, M., Cooper, D. R., Cymborowski, M., Grabowski, M., Wlodawer, A., Dauter, Z., Shabalin, I. G., Gilski, M., Rupp, B., Jaskolski, M. & Minor, W. (2021). *Protein Sci.* **30**, 115–124. 10.1002/pro.3959 PMC7537053 32981130

[bb6] Burley, S. K., Berman, H. M., Bhikadiya, C., Bi, C., Chen, L., Di Costanzo, L., Christie, C., Dalenberg, K., Duarte, J. M., Dutta, S., Feng, Z., Ghosh, S., Goodsell, D. S., Green, R. K., Guranović, V., Guzenko, D., Hudson, B. P., Kalro, T., Liang, Y., Lowe, R., Namkoong, H., Peisach, E., Periskova, I., Prlić, A., Randle, C., Rose, A., Rose, P., Sala, R., Sekharan, M., Shao, C., Tan, L., Tao, Y.-P., Valasatava, Y., Voigt, M., Westbrook, J., Woo, J., Yang, H., Young, J., Zhuravleva, M. & Zardecki, C. (2019). *Nucleic Acids Res.* **47**, D464–D474. 10.1093/nar/gky1004 PMC6324064 30357411

[bb7] Chen, W.-H., Du, L., Chag, S. M., Ma, C., Tricoche, N., Tao, X., Seid, C. A., Hudspeth, E. M., Lustigman, S., Tseng, C.-T. K., Bottazzi, M. E., Hotez, P. J., Zhan, B. & Jiang, S. (2014). *Hum. Vaccin. Immunother.* **10**, 648–658. 10.4161/hv.27464 PMC4130269 24355931

[bb8] Chou, C.-Y., Lai, H.-Y., Chen, H.-Y., Cheng, S.-C., Cheng, K.-W. & Chou, Y.-W. (2014). *Acta Cryst.* D**70**, 572–581. 10.1107/S1399004713031040 PMC7161584 24531491

[bb9] Chruszcz, M., Domagalski, M., Osinski, T., Wlodawer, A. & Minor, W. (2010). *Curr. Opin. Struct. Biol.* **20**, 587–597. 10.1016/j.sbi.2010.08.001 PMC3017768 20810277

[bb10] Clegg, W. (2021). *IUCrJ*, **8**, 4–11. 10.1107/S205225252001458X PMC7793003 33520238

[bb11] Cooper, D. R., Grabowski, M., Zimmerman, M. D., Porebski, P. J., Shabalin, I. G., Woinska, M., Domagalski, M. J., Zheng, H., Sroka, P., Cymborowski, M., Czub, M. P., Niedzialkowska, E., Venkata­ramany, B. S., Osinski, T., Fratczak, Z., Bajor, J., Gonera, J., MacLean, E., Wojciechowska, K., Konina, K., Wajerowicz, W., Chruszcz, M. & Minor, W. (2021). *Methods Mol. Biol.* **2199**, 209–236. 10.1007/978-1-0716-0892-0_13 PMC8019398 33125653

[bb12] Croll, T., Diederichs, K., Fischer, F., Fyfe, C., Gao, Y., Horrell, S., Joseph, A. P., Kandler, L., Kippes, O., Kirsten, F., Müller, K., Nolte, K., Payne, A., Reeves, M. G., Richardson, J., Santoni, G., Stäb, S., Tronrud, D., Williams, C. & Thorn, A. (2020). *bioRxiv*, 2020.10.07.307546.

[bb13] Croll, T. I., Williams, C. J., Chen, V. B., Richardson, D. C. & Richardson, J. S. (2021). *Biophys. J.* **120**, 1085–1096. 10.1016/j.bpj.2020.12.029 PMC7834719 33460600

[bb14] Ewers, M., Ioannidis, J. P. A. & Plesnila, N. (2021). *J. Clin. Epidemiol.* **130**, 143–146. 10.1016/j.jclinepi.2020.10.008 PMC7554475 33068714

[bb58] Faezov, B. & Dunbrack, R. L. Jr (2021). *bioRxiv*, 2021.02.14.431128.

[bb15] Grabowski, M., Cymborowski, M., Porebski, P. J., Osinski, T., Shabalin, I. G., Cooper, D. R. & Minor, W. (2019). *Struct. Dyn.* **6**, 064301. 10.1063/1.5128672 PMC6874509 31768399

[bb57] Grabowski, M., Langner, K. M., Cymborowski, M., Porebski, P. J., Sroka, P., Zheng, H., Cooper, D. R., Zimmerman, M. D., Elsliger, M.-A., Burley, S. K. & Minor, W. (2016). *Acta Cryst.* D**72**, 1181–1193. 10.1107/S2059798316014716 PMC5108346 27841751

[bb16] Grabowski, M., Niedzialkowska, E., Zimmerman, M. D. & Minor, W. (2016). *J. Struct. Funct. Genomics*, **17**, 1–16. 10.1007/s10969-016-9201-5 PMC4834271 26935210

[bb17] Hall-Swan, S., Antunes, D. A., Devaurs, D., Rigo, M. M., Kavraki, L. E. & Zanatta, G. (2021). *bioRxiv*, 2021.01.21.427315. 10.1016/j.compbiomed.2021.104943 PMC8518241 34717233

[bb18] Helliwell, J. R., Minor, W., Weiss, M. S., Garman, E. F., Read, R. J., Newman, J., van Raaij, M. J., Hajdu, J. & Baker, E. N. (2019). *IUCrJ*, **6**, 341–343. 10.1107/S2052252519005918 PMC6503929 31098014

[bb19] Henderson, J. A., Verma, N., Harris, R. C., Liu, R. & Shen, J. (2020). *J. Chem. Phys.* **153**, 115101. 10.1063/5.0020458 PMC7499820 32962355

[bb20] Iudin, A., Korir, P. K., Salavert-Torres, J., Kleywegt, G. J. & Patwardhan, A. (2016). *Nat. Methods*, **13**, 387–388. 10.1038/nmeth.3806 27067018

[bb21] Jaskolski, M. (2017). *Methods Mol. Biol.* **1607**, 549–563. 10.1007/978-1-4939-7000-1_22 28573588

[bb22] Jaskolski, M., Gilski, M., Dauter, Z. & Wlodawer, A. (2007). *Acta Cryst.* D**63**, 611–620. 10.1107/S090744490700978X 17452786

[bb23] Kabsch, W. (2010). *Acta Cryst.* D**66**, 125–132. 10.1107/S0907444909047337 PMC2815665 20124692

[bb24] Kolesov, G., Virnau, P., Kardar, M. & Mirny, L. A. (2007). *Nucleic Acids Res.* **35**, W425–W428. 10.1093/nar/gkm312 PMC1933242 17517776

[bb25] Kowiel, M., Brzezinski, D., Porebski, P. J., Shabalin, I. G., Jaskolski, M. & Minor, W. (2019). *Bioinformatics*, **35**, 452–461. 10.1093/bioinformatics/bty626 PMC6361236 30016407

[bb26] Lei, J., Kusov, Y. & Hilgenfeld, R. (2018). *Antiviral Res.* **149**, 58–74. 10.1016/j.antiviral.2017.11.001 PMC7113668 29128390

[bb27] Leslie, A. G. W. (2006). *Acta Cryst.* D**62**, 48–57. 10.1107/S0907444905039107 16369093

[bb28] Liebschner, D., Afonine, P. V., Moriarty, N. W., Poon, B. K., Chen, V. B. & Adams, P. D. (2021). *Acta Cryst.* D**77**, 48–61. 10.1107/S2059798320015879 PMC7787109 33404525

[bb29] Lubin, J. H., Zardecki, C., Dolan, E. M., Lu, C., Shen, Z., Dutta, S., Westbrook, J. D., Hudson, B. P., Goodsell, D. S., Williams, J. K., Voigt, M., Sarma, V., Xie, L., Venkatachalam, T., Arnold, S., Alvarado, L. H. A., Catalfano, K., Khan, A., McCarthy, E., Staggers, S., Tinsley, B., Trudeau, A., Singh, J., Whitmore, L., Zheng, H., Benedek, M., Currier, J., Dresel, M., Duvvuru, A., Dyszel, B., Fingar, E., Hennen, E. M., Kirsch, M., Khan, A. A., Labrie-Cleary, C., Laporte, S., Lenkeit, E., Martin, K., Orellana, M., de la Campa, M. O.-A., Paredes, I., Wheeler, B., Rupert, A., Sam, A., See, K., Zapata, S. S., Craig, P. A., Hall, B. L., Jiang, J., Koeppe, J. R., Mills, S. A., Pikaart, M. J., Roberts, R., Bromberg, Y., Hoyer, J. S., Duffy, S., Tischfield, J., Ruiz, F. X., Arnold, E., Baum, J., Sandberg, J., Brannigan, G., Khare, S. D. & Burley, S. K. (2020). *bioRxiv*, 2020.12.01.406637.

[bb30] Macnar, J. M., Szulc, N. A., Kryś, J. D., Badaczewska-Dawid, A. E. & Gront, D. (2020). *Biomolecules*, **10**, 461. 10.3390/biom10030461 PMC7175226 32188163

[bb31] Masmaliyeva, R. C. & Murshudov, G. N. (2019). *Acta Cryst.* D**75**, 505–518. 10.1107/S2059798319004807 PMC6503761 31063153

[bb32] McPherson, J. D. (2009). *Nat. Methods*, **6**, S2–S5. 10.1038/nmeth.f.268 19844227

[bb33] Meyer, P. A., Socias, S., Key, J., Ransey, E., Tjon, E. C., Buschiazzo, A., Lei, M., Botka, C., Withrow, J., Neau, D., Rajashankar, K., Anderson, K. S., Baxter, R. H., Blacklow, S. C., Boggon, T. J., Bonvin, A. M. J. J., Borek, D., Brett, T. J., Caflisch, A., Chang, C.-I., Chazin, W. J., Corbett, K. D., Cosgrove, M. S., Crosson, S., Dhe-Paganon, S., Di Cera, E., Drennan, C. L., Eck, M. J., Eichman, B. F., Fan, Q. R., Ferré-D’Amaré, A. R., Christopher Fromme, J., Garcia, K. C., Gaudet, R., Gong, P., Harrison, S. C., Heldwein, E. E., Jia, Z., Keenan, R. J., Kruse, A. C., Kvansakul, M., McLellan, J. S., Modis, Y., Nam, Y., Otwinowski, Z., Pai, E. F., Pereira, P. J. B., Petosa, C., Raman, C. S., Rapoport, T. A., Roll-Mecak, A., Rosen, M. K., Rudenko, G., Schlessinger, J., Schwartz, T. U., Shamoo, Y., Sondermann, H., Tao, Y. J., Tolia, N. H., Tsodikov, O. V., Westover, K. D., Wu, H., Foster, I., Fraser, J. S., Maia, F. R. N. C., Gonen, T., Kirchhausen, T., Diederichs, K., Crosas, M. & Sliz, P. (2016). *Nat. Commun.* **7**, 10882.

[bb34] Minor, W., Cymborowski, M., Otwinowski, Z. & Chruszcz, M. (2006). *Acta Cryst.* D**62**, 859–866. 10.1107/S0907444906019949 16855301

[bb35] Minor, W., Dauter, Z., Helliwell, J. R., Jaskolski, M. & Wlodawer, A. (2016). *Structure*, **24**, 216–220. 10.1016/j.str.2015.12.010 PMC4743038 26840827

[bb36] Miyakawa, T. (2020). *Mol. Brain*, **13**, 24. 10.1186/s13041-020-0552-2 PMC7033918 32079532

[bb37] Osipiuk, J., Azizi, S.-A., Dvorkin, S., Endres, M., Jedrzejczak, R., Jones, K. A., Kang, S., Kathayat, R. S., Kim, Y., Lisnyak, V. G., Maki, S. L., Nicolaescu, V., Taylor, C. A., Tesar, C., Zhang, Y.-A., Zhou, Z., Randall, G., Michalska, K., Snyder, S. A., Dickinson, B. C. & Joachimiak, A. (2021). *Nat. Commun.* **12**, 743. 10.1038/s41467-021-21060-3 PMC7854729 33531496

[bb38] Otwinowski, Z. & Minor, W. (1997). *Methods Enzymol.* **276**, 307–326. 10.1016/S0076-6879(97)76066-X 27754618

[bb39] Parks, J. M. & Smith, J. C. (2020). *N. Engl. J. Med.* **382**, 2261–2264. 10.1056/NEJMcibr2007042 32433861

[bb40] Pearce, N. M., Krojer, T. & von Delft, F. (2017). *Acta Cryst.* D**73**, 256–266. 10.1107/S2059798317003412 PMC5349438 28291761

[bb41] Raczynska, J. E., Shabalin, I. G., Minor, W., Wlodawer, A. & Jaskolski, M. (2018). *Drug Resist. Updat.* **40**, 1–12. 10.1016/j.drup.2018.08.001 PMC6260963 30466711

[bb42] Rahman, F., Tabrez, S., Ali, R., Alqahtani, A. S., Ahmed, M. Z. & Rub, A. (2021). *J. Tradit. Complement. Med.* **11**, 173–179. 10.1016/j.jtcme.2021.01.006 PMC7825826 33520682

[bb43] Rupp, B. (2009). *Biomolecular Crystallography: Principles, Practice, and Application to Structural Biology*. New York: Garland Science.

[bb44] Sedova, M., Jaroszewski, L., Alisoltani, A. & Godzik, A. (2020). *Bioinformatics*, **36**, 4360–4362. 10.1093/bioinformatics/btaa550 PMC7314196 32470119

[bb45] Shabalin, I., Dauter, Z., Jaskolski, M., Minor, W. & Wlodawer, A. (2015). *Acta Cryst.* D**71**, 1965–1979. 10.1107/S139900471500629X PMC4556316 26327386

[bb46] Singh, J. (2011). *J. Pharmacol. Pharmacother.* **2**, 138–139. 10.4103/0976-500X.81919 PMC3127351 21772785

[bb47] Touw, W. G., Joosten, R. P. & Vriend, G. (2016). *J. Mol. Biol.* **428**, 1375–1393. 10.1016/j.jmb.2016.02.002 26869101

[bb48] Waman, V. P., Sen, N., Varadi, M., Daina, A., Wodak, S. J., Zoete, V., Velankar, S. & Orengo, C. (2020). *Brief. Bioinform.*, bbaa362. 10.1093/bib/bbaa362 PMC7799268 33348379

[bb49] Ward, J. H. (1963). *J. Am. Stat. Assoc.* **58**, 236–244.

[bb50] Winter, G., Waterman, D. G., Parkhurst, J. M., Brewster, A. S., Gildea, R. J., Gerstel, M., Fuentes-Montero, L., Vollmar, M., Michels-Clark, T., Young, I. D., Sauter, N. K. & Evans, G. (2018). *Acta Cryst.* D**74**, 85–97. 10.1107/S2059798317017235 PMC5947772 29533234

[bb51] Wlodawer, A., Dauter, Z., Porebski, P. J., Minor, W., Stanfield, R., Jaskolski, M., Pozharski, E., Weichenberger, C. X. & Rupp, B. (2018). *FEBS J.* **285**, 444–466. 10.1111/febs.14320 PMC5799025 29113027

[bb52] Wlodawer, A., Dauter, Z., Shabalin, I. G., Gilski, M., Brzezinski, D., Kowiel, M., Minor, W., Rupp, B. & Jaskolski, M. (2020). *FEBS J.* **287**, 3703–3718. 10.1111/febs.15366 PMC7276724 32418327

[bb53] Wlodawer, A., Minor, W., Dauter, Z. & Jaskolski, M. (2013). *FEBS J.* **280**, 5705–5736. 10.1111/febs.12495 PMC4080831 24034303

[bb54] Zheng, H., Hou, J., Zimmerman, M. D., Wlodawer, A. & Minor, W. (2014). *Exp. Opin. Drug. Discov.* **9**, 125–137. 10.1517/17460441.2014.872623 PMC4106240 24372145

[bb55] Zheng, H., Porebski, P. J., Grabowski, M., Cooper, D. R. & Minor, W. (2017). *Methods Mol. Biol.* **1607**, 643–665. 10.1007/978-1-4939-7000-1_27 PMC5587190 28573593

[bb56] Zimmerman, M. D., Grabowski, M., Domagalski, M. J., Maclean, E. M., Chruszcz, M. & Minor, W. (2014). *Methods Mol. Biol.* **1140**, 1–25. 10.1007/978-1-4939-0354-2_1 PMC4086192 24590705

