# Supplementary material for: Rapid response to emerging biomedical challenges and threats
Source: IUCrJ. 2021 Mar 26;8(Pt 3):395–407. doi: 10.1107/S2052252521003018 (PMC8086160; doi:10.1107/S2052252521003018)
Supplement: Supplementary file 1 [file m-08-00395-sup1.pdf]

IUCrJ

Volume 8 (2021)

Supporting information for article:

Rapid response to emerging biomedical challenges and threats

Marek Grabowski, Joanna M. Macnar, Marcin Cymborowski, David R. Cooper, Ivan G. Shabalin, Miroslaw Gilski, Dariusz Brzezinski, Marcin Kowiel, Zbigniew Dauter, Bernhard Rupp, Alexander Wlodawer, Mariusz Jaskolski and Wladek Minor

Data collection and refinement statistics for project **5s32** crystal **crystal1**

model **/home2/marcel/HKL/data/rerefinement/test2/5s32/structure_mr/build_model_1/hkl_import.pdb**

**Data collection**	
Resolution (Å)	88.12 - 1.17 (1.23 - 1.17)
Wavelength (Å)	0.91188
Space group	P43
a, b, c (Å)	88.12, 88.12, 39.08
α, β, γ (°)	90, 90, 90
Completeness (%)	98.4
Reflections used	100790
<I> / <Sigma I>	3.4
Redundancy	6.3 (5.2)
Rmerge	0.532 !
Rpim	0.231 (5.759) !
CC1/2 last shell	0.36
Wilson B factor (Å²)	11.2
**Refinement**	
Rwork / Rfree	0.201 / 0.236
Resolution (Å)	88.12 - 1.17
Reflections all	88766
Reflections for Rfree	4499, 5.1%
Bond lengths rmsd (Å)	0.010
Bond angles rmsd (°)	1.12
Mean B value (Å²)	18
Number of protein atoms	2581
Mean B value for protein atoms (Å²)	15
Number of water atoms (expected)	472 (520)
Mean B value for water atoms (Å²)	29
Number of ligand/ion atoms	16
Mean B value for ligand/ion atoms (Å²)	24
Clashscore	3.07
Clashscore percentile (100)	74.8
Rotamer outliers (<1%)	0.70
Ramachandran outliers (<0.2%)	0.00
Ramachandran favored (>98%)	99.09
Residues with bad bonds (<0%)	0.00
Residues with bad angles (<0.1%)	0.45
MolProbity score	1.10

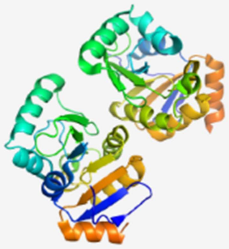

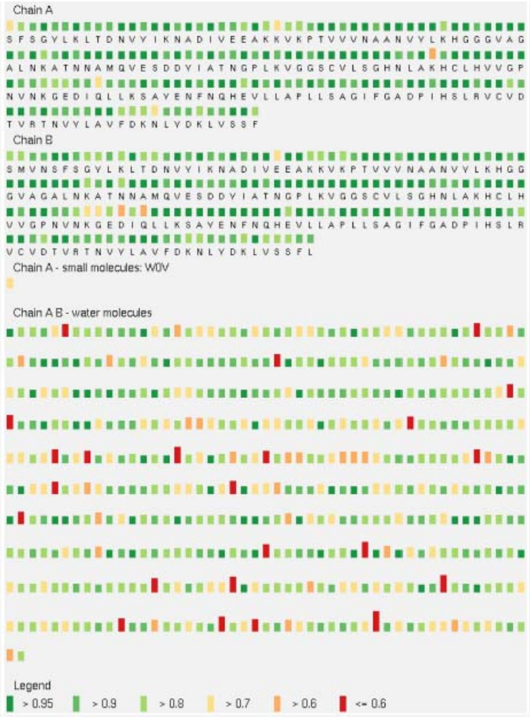

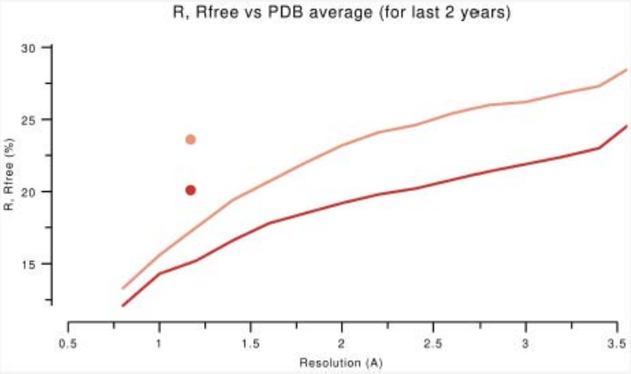

Credits:

HKL-3000
"Processing of X-ray Diffraction Data Collected in Oscillation Mode"
Z.Otwinowski, W.Minor
Methods in Enzymology, Volume 276: Macromolecular Crystallography, part A, p307-326 (1997)
"HKL-3000: the integration of data reduction and structure solution - from diffraction images to an initial model in minutes"
W.Minor, M.Cymborowski, Z.Otwinowski, M.Chruszcz
Acta Cryst. D62: 859-866 (2006)

CCP4 suite
"Overview of the CCP4 suite and current developments"
M.D.Winn et al.
Acta. Cryst. D67, 235-242 (2011)

Refmac5
"REFMAC5 for the refinement of macromolecular crystal structures"
G.N.Murshudov, P.Skubak, A.A.Lebedev, N.S.Pannu, R.A.Steiner, R.A.Nicholls, M.D.Winn, F.Long and A.A.Vagin
Acta Cryst. D67, 355-367 (2011)

Coot
"Features and Development of Coot"
P.Emsley, B.Lohkamp, W.Scott, and K.Cowtan
Acta Cryst. D66, 486-501 (2010)

Figure S1 The complete structure quality report from HKL-3000

Data Collection

Overall

ID #	Resolution (High)	Resolution (Low)	Percent Possible (Observed)	R Merge I (Observed)	CC (Half)	Net I Over Average Sigma (I)	Redundancy	Number Reflections (All)	Number Reflections (Observed)	Observed Criterion Sigma (F)	Observed Criterion Sigma (I)	B (Isotropic) From Wilson Plot
1	3.31	80.253	100	0.091	1	0.018	25.6		22105			

Highest Resolution Shell

ID #	Resolution (High)	Resolution (Low)	Percent Possible (All)	Percent Possible (Observed)	R Merge I (Observed)	Rpim I (All)	CC (Half)	Mean I Over Sigma (Observed)	Redundancy	Number Unique Reflections (All)
1	3.31	3.4			3.9	0.77	0.3	0.5		

Dataset statistics

Metric	Value	Source
Cell dimensions	**a:** 156.411 Å **b:** 156.411 Å **c:** 116.205 Å<br>**α:** 90° **β:** 90° **γ:** 90°	Depositor
Number of reflections	22065	Xtriage(Phenix)
Test set size	4.93%	EDS
Data completeness	100%	EDS
EDS resolution	3.31Å - 80.11Å	EDS
<I/σ(I)>	1.1	Xtriage(Phenix)
Twinning statistics	$\langle \lvert L \rvert \rangle = 0.39$, $\langle L^2 \rangle = 0.21$	Xtriage(Phenix)
Spacegroup	$P\ 4_2\ 2_1\ 2$	Depositor
Possible (pseudo-) translation	The largest off-origin peak in the Patterson function is 3.12% of the height of the origin peak. No significant pseudotranslation is detected.	Xtriage(Phenix)
Wilson B	160 Å^2	Xtriage(Phenix)

Figure S2 The discrepancy in the reported <I/sigma(I)> values between various wwPDB affiliates for the structure 6ZH9. The top of the figure is from the RCSB PDB and the bottom is from the PDBe

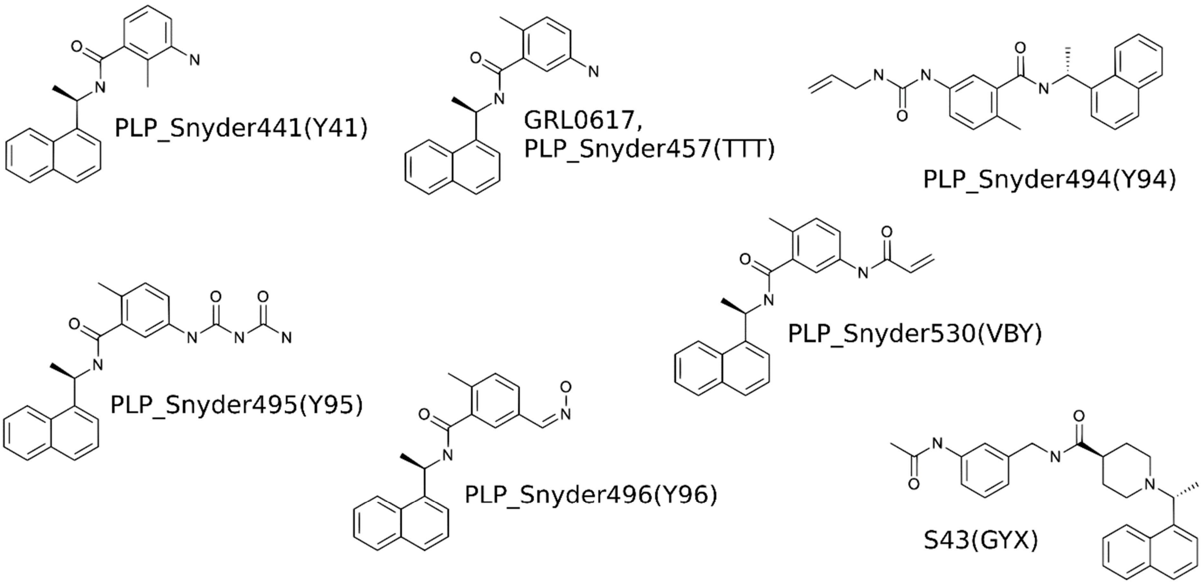

Figure S3 Chemical structures of unique inhibitors bound in PLpro structures. The compounds are labeled with the inhibitor name as reported in the PDB title/header. The PDB ligand codes are in parentheses.

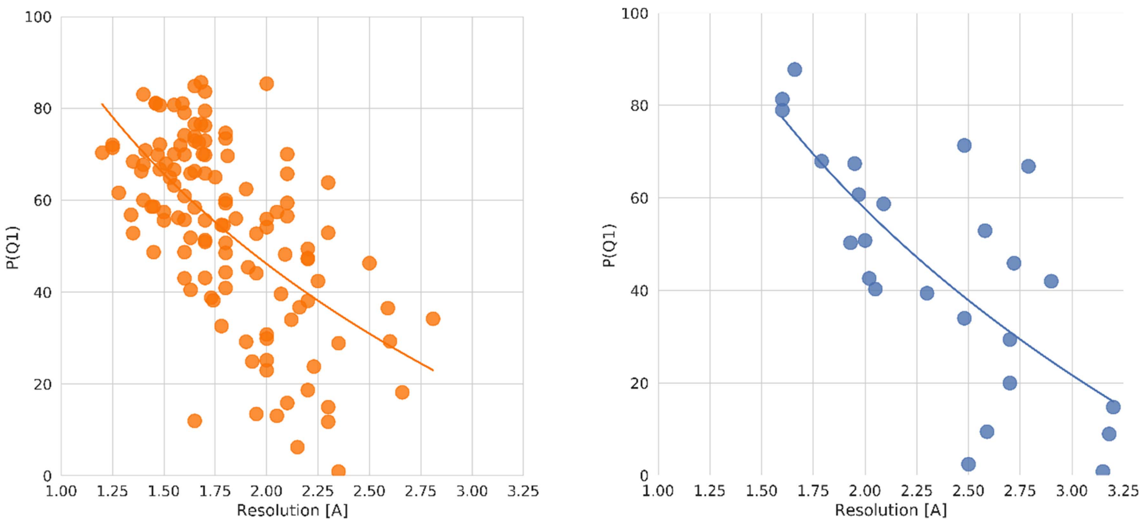

Figure S4 Plots showing the overall structure quality P(Q1) as a function of structure resolution for the main proteases (orange) and papain-like proteases (blue). It is clear that resolution cannot be the only parameter used to select the best structural model for subsequent studies, like docking or other computational studies. The lines are only for eye-guidance.

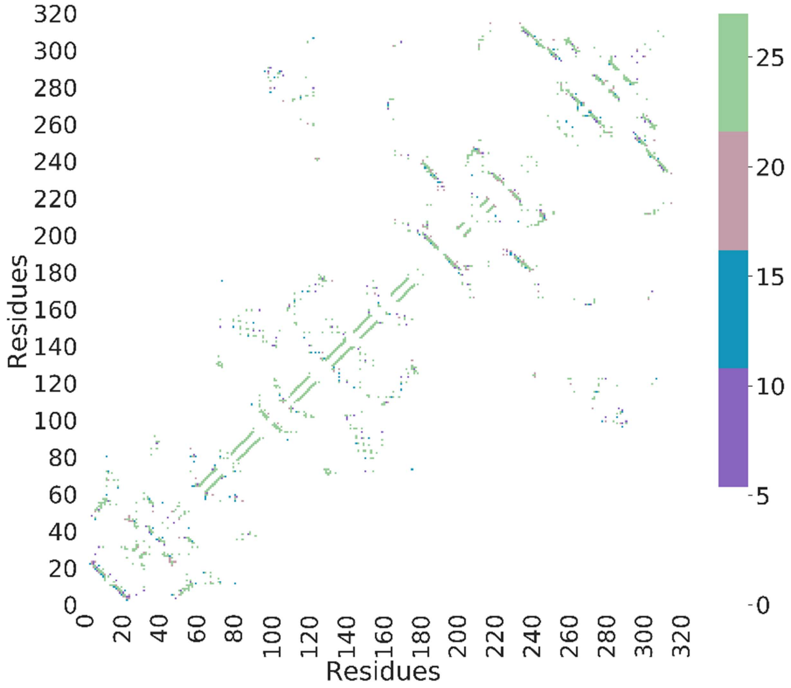

Figure S5 Contact map of residues from PLpro PDB deposits that indicates in how many models a given pair of residues are closer than 4.0 Å. Contacts between two consecutive residues are excluded.

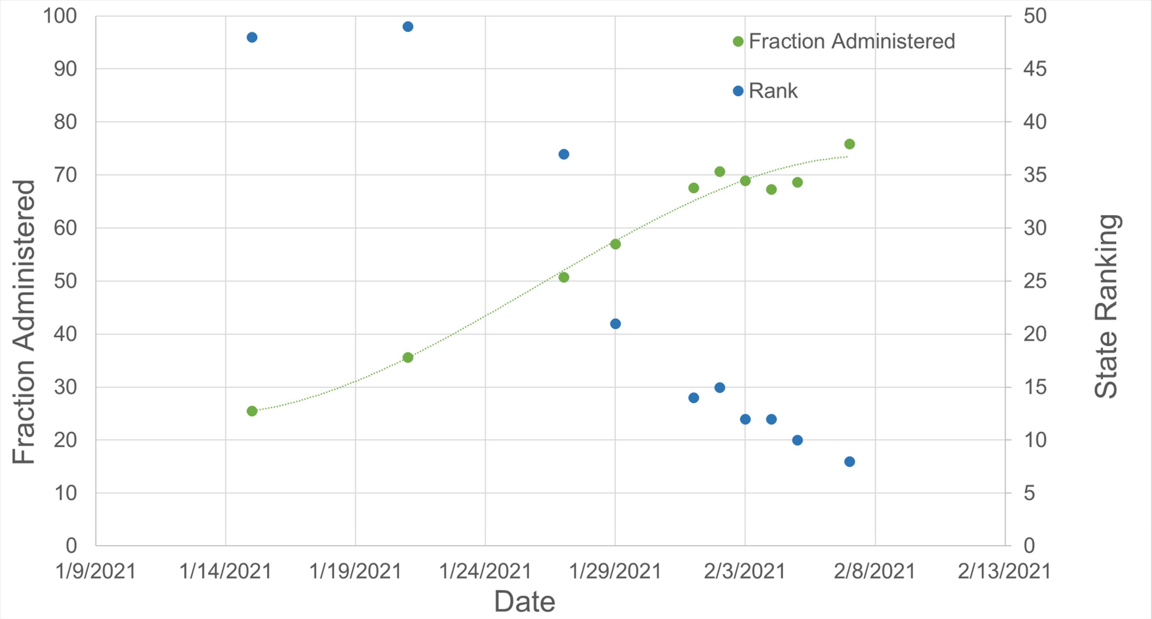

Figure S6 Vaccine Distribution and Administration in the Commonwealth of Virginia, USA. The blue circles represent the rank of efficiency defined as the fraction of distributed vaccine doses that were administered on a given date. Green circles represent the efficiency, which exceeded 80% as of 2/9/2021. On that date, Virginia ranked number one in efficiency among states that had administered more than 1 million vaccine doses. As the vaccination data are updated regularly, the current Commonwealth of Virginia efficiency is presented at https://virginia.bioreproducibility.org. Source: https://www.beckershospitalreview.com/public-health/states-ranked-by-percentage-of-covid-19-vaccines-administered.html and New York Times.
